# Preconception laparoscopic transabdominal cervical cerclage for the prevention of midtrimester pregnancy loss and preterm birth: a single centre experience

**Published:** 2019-03

**Authors:** E Saridogan, OP O’Donovan, AL David

**Affiliations:** Women’s Health, Elizabeth Garrett Anderson Wing, University College London Hospital, 235 Euston Road, London, NW1 2BU, United Kingdom;; Institute for Women’s Health, University College London, 86-96 Chenies Mews, London WC1E 6HX, United Kingdom;; St Michael’s Hospital, Southwell Street, Bristol BS28EG, United Kingdom;; NIHR University College London Hospitals Biomedical Research Centre, Maple House, 149 Tottenham Court Road, London W1T 7DN, United Kingdom.

**Keywords:** Cervical cerclage, transabdominal, transabdominal cerclage preterm birth, second trimester pregnancy loss, laparoscopy

## Abstract

**Background:**

A recent Cochrane review concluded that cervical cerclage reduces preterm birth before 37, 34 and 28 weeks of gestation and also probably reduces the risk of perinatal death. Transabdominal cerclage was developed for a subgroup in whom transvaginal cerclage had failed or was not possible. This approach appeared more effective in improving foetal survival rates or obstetric outcomes. Most commonly transabdominal cervical cerclage is placed at laparotomy (open transabdominal cerclage), but with the advance of minimal access techniques, laparoscopic transabdominal cervical cerclage is replacing the traditional open operation. The objective of this prospective case series is to explore the outcomes of pre-conception laparoscopic transabdominal cerclage procedures.

**Method:**

Data was prospectively collected from 54 women at high risk of second trimester miscarriage and preterm delivery due to cervical insufficiency undergoing pre-conception laparoscopic transabdominal cerclage by a single operator. This included demographics, obstetric and gynaecological history (including previous cervical cerclage procedures), surgical complication rates, conception and subsequent pregnancy outcomes.

**Results:**

There were 36 pregnancies progressing beyond the first trimester with a “take home baby” rate of 89% (32/36), a live birth rate of 92% (33/36) and neonatal survival rate of 97% (32/33). The mid-trimester loss (MTL) rate was 8% (3/36) with delivery rates after 37 weeks of 75% (27/36) and between 34 -37 weeks of 8% (3/36) and 23-34 weeks of 8% (3/36).

**Conclusions:**

Our prospective case series provides further evidence that laparoscopic transabdominal cerclage (TAC) is feasible, safe and effective when transvaginal cerclage fails or is not possible.

## Introduction

Cervical cerclage is used for the management of women considered to be at high risk of midtrimester loss (MTL) and spontaneous preterm birth (PTB) (Ades et al., 2015; NICE, 2015; Alfirevic et al., 2017). Sonographic cervical shortening in women with a previous PTB, MTL or cervical surgery is commonly used as an indication to place a transvaginal (TV) cervical cerclage, using either a McDonald (low vaginal) or Shirodkar (high vaginal) technique. Meta-analysis of trials using individual patient-level data shows that in this population diagnosed as having an ‘insufficient cervix’, transvaginal cervical cerclage significantly reduces delivery before 35 weeks of gestation (Berghella et al., 2005).

A recent Cochrane review concluded that cervical cerclage reduces PTB before 37, 34 and 28 weeks of gestation and also probably reduces the risk of perinatal death (Alfirevic et al., 2017). Not all transvaginal cervical cerclages are successful in achieving a livebirth however. Factors affecting success may include the type of suture material used and the height of the cerclage in relation to the external cervical os. A large retrospective cohort study found a perinatal loss rate after transvaginal cervical cerclage of 15% in women receiving braided suture material, which was significantly higher than the rate of 5% in those receiving monofilament (Kindinger et al., 2016). The type of suture material is now being investigated in a large randomized control trial in the UK. In another retrospective cohort study, increasing absolute cerclage height was associated with a reduction in spontaneous PTB (Cook et al., 2017).

Transabdominal cerclage was developed for a subgroup of women in whom transvaginal cerclage had failed or was not possible. This approach appeared more effective in improving foetal survival rates (Debbs et al., 2007) or obstetric outcomes (Davis et al., 2000) in this highly selected subgroup. Most commonly transabdominal cervical cerclage is placed at laparotomy (open Transabdominal Cerclage, TAC). Some studies have suggested that compared to first trimester open TAC, pre-conceptual open TAC is more successful in preventing repeat spontaneous MTL and PTB, and is associated with less surgical and pregnancyrelated morbidity (Whittle et al., 2009; Dawood and Farquharson, 2016). With the advance of minimal access techniques, laparoscopic transabdominal cervical cerclage (laparoscopic TAC) is replacing the traditional open operation, with the advantages of avoidance of a large abdominal incision including shorter hospital stay, faster recovery and better cosmesis. Initial case reports and series (El-Nashar et al., 2013; Moawad et al., 2018) suggested that laparoscopic TAC achieves similar outcomes to open TAC. Open TAC procedures can be performed towards the end of the first trimester after a dating scan has confirmed a healthy fetus. In contrast, most laparoscopic TAC procedures are reported to have been placed pre-conception due to concerns that laparoscopic manipulation of the pregnant uterus may increase the risk of post-procedure pregnancy loss (Whittle et al., 2009; Tulandi, et al., 2014; Moawad et al., 2018). There were more conversions to laparotomy and cerclage failures when the procedure was performed during pregnancy in the series by Whittle et al. (2009) in addition to the perioperative foetal loss rate, which was 6.4% (2/31).

We started replacing the open TAC with a laparoscopic TAC approach in 2004, and prospectively audited our outcomes in accordance with National Institute for Health and Care Excellence (NICE) new interventional procedures guidelines. In this report we present the outcomes of the laparoscopic TAC procedures over a 13 year period.

## Methods

Data were collected prospectively on patients undergoing laparoscopic TAC procedure between August 2004 and December 2017 encompassing demographics, gynaecological and obstetric history; including any operations involving the cervix, conception rate and outcomes of all pregnancies, history and type of any previous cervical cerclage procedures. Subsequent to laparoscopic TAC, all data on surgical complications related to placement, conception rates and pregnancy outcomes were recorded. When the patient remained under the care of our hospital data was extracted from their case notes, otherwise we contacted the patient or referring doctor via a letter or by phone.

Such service evaluation projects do not require ethical review by a National Health Service (NHS) or Social Care Research Ethics Committee or management permission through the NHS Research and Development Office. Under these circumstances, there was no need to submit applications to the NHS Research Ethics Committee or NHS/Health and Social Care Research and Development office (www.hra.nhs.uk).

**Figure 1:**
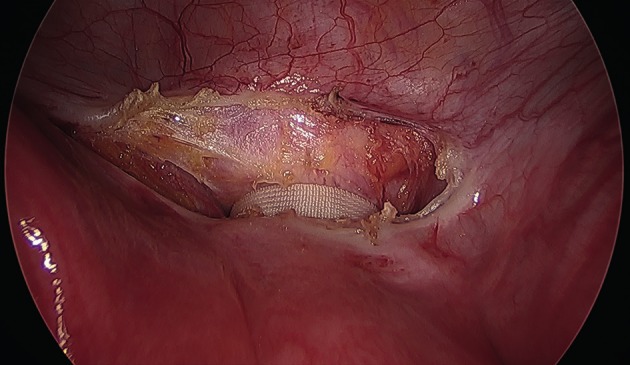


**Figure 2:**
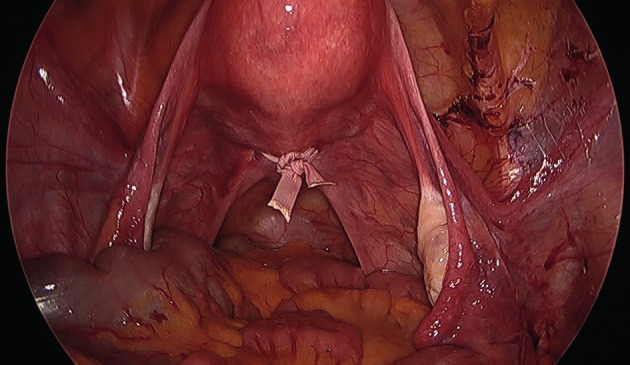


All laparoscopic TAC procedures were carried out pre-conception, using a technique as previously published by this group (Gibb and Saridogan, 2016). After general anaesthesia, the patient was positioned in dorsal lithotomy and a Foley’s urinary catheter was inserted. After a vaginal speculum and bimanual examination, the cervix was grasped with a vulsellum and a simple uterine manipulator (Spackman’s cannula) was inserted. A 10 mm 0 degree laparoscope was inserted through an intraumbilical port and two additional 5 mm lateral ports inserted under direct vision at the level of the umbilicus lateral to the midclavicular line. The uterovesical peritoneum was opened at the isthmic level using scissors with monopolar diathermy or ultrasonic scalpel, and extended slightly laterally to expose the uterine vessels on both sides. There was usually no need to reflect the bladder, except in circumstances where the bladder was pulled up onto the anterior uterine wall following, for example, a caesarean section. A 5 mm Mersilene® tape suture with a curved blunt needle (Ethicon, Somerville, NJ, USA) was then passed in the anteroposterior direction between the uterine vessels and the cervicoisthmic junction, emerging through the posterior leaf of the broad ligament approximately 1 cm above the uterosacral ligament. The same approach was then repeated on the contralateral side and the knot was tied posterior to the cervicoisthmic junction. Care was taken to lay the tape flat on the uterus (Figure 1) and to cut the ends leaving only 1-2cm (Figure 2). More recently we have adapted our technique by straightening the needle to assist passing it in the antero-posterior direction. The uterovesical peritoneum was not usually closed. The bladder catheter was removed at the end of the procedure and patients were discharged home on the same day after having eaten and passed urine. The patients were asked to report back when they became pregnant.

## Results

### Study population

Fifty-four preconception laparoscopic cerclage procedures were successfully performed during the study period. The mean age of the women was 36.0 years (range 23-44 years). Eight of these women were nulligravid but had a history of 1-3 cone biopsies, large loop excision of the transformation zone (LLETZ) procedures, or trachelectomy; five of these for early invasive cervical cancer. In each case the women had a very short cervix or no visible ectocervix, hence a transvaginal cerclage was considered impossible.

Forty-six patients had previously been pregnant (range gravida 1-10), with 75 second trimester losses in total between them; 16 women had no living children and 30 had at least one living child; 16 women had had a previous MTL or PTB (23-34 weeks) despite a transvaginal cervical cerclage (Table I). 16 women had a history of at least one termination of pregnancy (TOP), four in the first trimester, three in the second trimester and nine undeclared.

**Table I:** — Past medical history of study population.

	Total women	Poor obstetric outcome	Cervical surgery	Both
Total women	54	30	16	8
Previous transvaginal cerclage	27	24	0	3
Previous termination of pregnancy	16	14	1	1
Uterine abnormalities	1^1^	1^1^	0	0
Any previous delivery 23-34 weeks gestation	21	17	0	4
Previous delivery 23-34 weeks gestation despite transvaginal cerclage	16	15	0	1
Previous cervical surgery	24	0	16	8

^1^Unicornuate uterus

### Surgical outcome

All procedures were performed by the same surgeon (ES). There were no significant intraoperative or postoperative complications. All patients went home on the same day as the procedure except one who had a planned concurrent laparoscopic myomectomy.

### Postoperative obstetric outcome

Of 54 women having laparoscopic TAC procedures, four women were lost to follow-up (2 were from abroad and 2 could not be contacted). Over a third of procedures (21 women, 39%) were performed in the most recent two years of the study period. Ten procedures were performed within the last year, and of these cases, one woman has delivered a term baby, one is currently pregnant in the first trimester, two in the second trimester and the other seven have not yet conceived (one is not actively trying). Of the 40 women successfully followed-up whose procedures was performed at least 1 year ago, 33 have conceived giving a fertility rate of 83%. 36 women had 42 pregnancies after the laparoscopic TAC procedure, with 40 pregnancies progressing beyond the first trimester. There were 2 first trimester miscarriages (5%) and there are four ongoing pregnancies (three second trimester and one third trimester), hence 36 pregnancies with known outcome have progressed beyond the first trimester. The outcomes of these pregnancies are described in Table II and III.

**Table II:** — Pregnancy outcomes.

Previous history	Gestation at delivery/pregnancy loss (weeks)					Perinatal outcome	
All	12-23 (%)	23-34 (%)	34-37 (%)	37+ (%)	Live births (%)	Neonatal survival (%)
All	36	3 (8)	3 (8)	3 (8)	27 (75)	33 (92)	32 (97)
Poor obstetric history	22	2 (9)	2 (9)	1 (5)	17 (77)	20 (91)	19 (95)
Cervical surgery	8	0 (0)	1 (13)	1 (13)	6 (75)	8 (100)	8 (100)
Both	6	1 (17)	0	1 (17)	4 (67)	5 (83)	5 (83)

**Table III:** — Outcome of pregnancies delivered 12-37 weeks gestation

	Outcome of pregnancies delivered 12-37 weeks gestation
1	Spontaneous rupture of membranes at 24 weeks, posterior colpotomy to remove suture, induction of labour (suspected chorioamnionitis), neonatal death.
2	Elective caesarean section at 36+6 weeks.
3	Caesarean section at 27 weeks due to severe pre-eclampsia and intrauterine growth restriction.
4	Caesarean section at 34 weeks (no other information available).
5	Elective caesarean section at 35 weeks for placenta praevia (previous caesarean section), 3.5 litre postpartum haemorrhage.
6	Emergency caesarean section 30+2 for pre-term labour.
7	18 week miscarriage (hysterotomy)
8	15 week miscarriage (went on to have term pregnancy)
9	19 week miscarriage

Thirty-four of the 37 women (92%) with known outcomes who fell pregnant following laparoscopic TAC have taken home a baby. All five of those who had never been pregnant before were delivered at term; 29 (91%) of the other 32 women had live births, and the remaining 3 were late miscarriages (see Table III).

## Discussion

This study reports the prospective experience of a laparoscopic TAC procedure clinical service over the last 13 years. We have demonstrated favorable outcomes in the majority of women who underwent this procedure. Live birth and neonatal survival rates of 92% and 97% are remarkable in this group despite their high risk of MTL and PTB due to their complex gynaecologic and/or obstetric histories.

The strengths of our study are its prospective nature, stringent patient selection and performance of operations by a single surgeon. We collected standardized data on past obstetric and gynaecological history, and monitored the outcome of subsequent pregnancies at regular intervals. We only included women with appropriate obstetric history and/or cervical surgery causing absent ectocervix or short cervix.

The main limitation is lack of a control group, particularly for those who had not been pregnant before. It would be difficult to randomize women with a history of failed transvaginal cerclage, but it may be possible to collect data from women who choose to have expectant management instead of laparoscopic TAC following cervical surgery causing absent ectocervix or short cervix.

These findings are replicated elsewhere in recent observational studies of laparoscopic TAC, with Huang et al. (2016) reporting a live birth rate of 96% and neonatal survival rate of 100% and Ades et al. (2018) a perinatal survival rate of 98.4%. Neonatal survival was 89% in the laparoscopic arm of Moawad et al. (2018) systematic review comparing open and laparoscopic TAC.

The basic premise behind cervical cerclage is to delay delivery of the fetus to a gestational age where neonatal outcomes are improved. Major milestones in gestational age are 34 and 37 weeks, at which we achieved delivery rates of 83% and 75% respectively, supporting the success of this technique. Again this is echoed in the other studies with data from Ades et al. (2018) showing 86% delivery rate past 34 weeks and 52% at term, and Huang et al. (2016).

MTL affects 2-3% of pregnancies (Wyatt et al., 2005) and PTB occurs in 5%. They are thought to have similar aetiology including infection, cervical weakness (insufficiency), congenital uterine anomalies, antiphospholipid syndrome and placental insufficiency. Cervical weakness may be due to a number of factors affecting the structural integrity and ability to prevent against ascending infection (Lee et al., 2008) such as previous cervical surgery for cervical intraepithelial neoplasia (CIN) and early invasive cervical cancer (cone biopsy, LLETZ), cervical lacerations due to traumatic deliveries and forced cervical dilatation for pregnancy terminations, and increasingly full dilatation Caesarean section is being recognized as a risk factor (Watson et al., 2017). In our highly selected group of women there were two main surgical indications; those with poor obstetric history many of whom had a previous failed transvaginal cerclage, and those with a history of cervical surgery with no visible ectocervix or short cervix, in whom a transvaginal cerclage was deemed not feasible. There was a small group who had history of both. The reasons and mechanisms of MTL and PTB may be considered different in these groups, and one might anticipate a higher success rate in the cervical surgery group. However, our results, as well as those of others, suggest that laparoscopic TAC is similarly successful in both groups. It is possible that TAC may act through maintenance of the cervical mucus plug and prevention of ascending infection as well as its mechanical support.

Cervical cerclage procedure is usually carried out during pregnancy via the transvaginal route. There are however circumstances in which the transvaginal approach may not be possible due to previous cervical surgery or trauma which leads to absence of ectocervical tissue in which to place the cerclage. Transabdominal approach is the logical alternative in this group of women. In our series, some women had not been pregnant before but were deemed to be at risk of MTL or PTL. Following counselling about this risk they choose to undergo the laparoscopic TAC procedure. An alternative approach would be expectant management, but in our experience many women prefer to know that they have a cerclage before embarking on a pregnancy and a randomized controlled trial in this group would be difficult.

Some women experience MTL or PTB despite a TV cerclage procedure. As with our cases, this group of women form the majority of cases included in the published literature. Amongst the women in our series, there was a striking number with history of MTLs and PTBs despite TV cerclage procedure. Transabdominal placement of the cerclage suture appears to be more successful than further attempts of transvaginal cerclage in these situations (Davis et al., 2000).

The third group of women who had had term deliveries before, had a history of a cervical surgery which may have resulted in weakness of the cervix in subsequent pregnancies. The laparoscopic TAC procedure appears to have worked effectively for this group as well with live birth rate of over 90%. There were six women who delivered preterm in our series. Failure of laparoscopic TAC in a small proportion of these women may be a reflection of the more complex underlying pathology in some women with a history of MTL and PTB that may not be resolved by cervical cerclage. Three PTBs were due to obstetric complications (placenta praevia, preeclampsia or intrauterine growth restriction) and one delivered electively at 36+6 weeks gestation without pregnancy complication (presumably for non-medical reasons e.g. capacity issues).

Published series of laparoscopic TAC report very low complication rates (Ades et al., 2018; Moawad et al., 2018). We have not had any surgical complications in 54 operations. The procedure is relatively simple but still requires appropriate skills in laparoscopic surgery and suturing; published series are likely to come from groups with this expertise. Maintenance of this approach and performance of laparoscopic TAC in centres where the necessary expertise is available is likely to preserve the low complication rates.

## Conclusion

Our prospective case series provides evidence that laparoscopic TAC is feasible, safe and effective when TV cerclage fails or is not possible. Comparative trials are likely to be difficult, but prospective data collection and development of national or international registries are likely to produce further evidence on its use for wider indications.
